# Plasmid diversity in arctic strains of *Psychrobacter* spp.

**DOI:** 10.1007/s00792-013-0521-0

**Published:** 2013-03-12

**Authors:** Lukasz Dziewit, Adrian Cegielski, Krzysztof Romaniuk, Witold Uhrynowski, Antoni Szych, Pawel Niesiobedzki, Magdalena J. Zmuda-Baranowska, Marek K. Zdanowski, Dariusz Bartosik

**Affiliations:** 1 Department of Bacterial Genetics, Institute of Microbiology, Faculty of Biology, University of Warsaw, Miecznikowa 1, 02-096 Warsaw, Poland; 2 Department of Antarctic Biology, Institute of Biochemistry and Biophysics, Polish Academy of Sciences, Ustrzycka 10/12, 02-141 Warsaw, Poland

**Keywords:** *Psychrobacter*, Plasmid, Host range, Mobilization for conjugal transfer, *ahpC*

## Abstract

Six strains of *Psychrobacter* spp. isolated from guano of little auks collected on Spitsbergen island (Arctic) carried nine plasmids that were fully sequenced. These replicons (ranging in size from 2917 to 14924 bp) contained either *repA* (ColE2-type) or *repB* (iteron-type) replication systems of a relatively narrow host range, limited to *Psychrobacter* spp. All but one of the plasmids carried predicted mobilization for conjugal transfer systems, encoding relaxases of the MOB_Q_, MOB_V_ or MOB_P_ families. The plasmids also contained diverse additional genetic load, including a type II restriction-modification system and a gene encoding a putative subunit C of alkyl hydroperoxide reductase (AhpC)—an antioxidant enzyme and major scavenger of reactive oxygen species. Detailed comparative sequence analyses, extended to all plasmids identified so far in psychrophilic bacteria, distinguished groups of the most ubiquitous replicons, which play a key role in horizontal gene transfer in cold environments.

**Electronic supplementary material:**

The online version of this article (doi:10.1007/s00792-013-0521-0) contains supplementary material, which is available to authorized users.

## Introduction

The polar regions occupy over 20 % of the Earth’s terrestrial surface. The High Arctic regions, such as the Svalbard Archipelago, are the coldest and most extreme. A greater part (58 %) of the Svalbard Archipelago is permanently covered in snow and ice, and this region also experiences low humidity, strong winds and cyclical, long periods of daylight and darkness (Thomas et al. 2008). Spitsbergen is the largest island of the Svalbard Archipelago in Norway. It lies within the north Arctic tundra and borders the Arctic Ocean, the Norwegian Sea and the Greenland Sea. The Spitsbergen climate is extremely harsh, with a mean annual air temperature of −4.4 °C, although due to the frequent winds, usually exceeding 30 m s^−1^, the perceived temperature can be even lower. The annual amplitude of temperatures is 49.4 °C, with a minimum of −35.9 °C, and a maximum of 13.5 °C (Nowosielski 2004; Przybylak and Araźny 2006).

Bacteria that tolerate low temperatures are the dominant life forms in polar ecosystems. These cold-adapted microorganisms can be divided into two overlapping ecological groups: (1) psychrophiles, whose cardinal growth temperatures (minimum, optimum and maximum) are 0, 15 and 20 °C, respectively, and (2) psychrotrophs (or psychrotolerants), that grow in a broader range of temperatures, between 0 and about 30 °C (Morita 1975). Psychrophiles and psychrotolerant microorganisms are subpopulations of bacteria inhabiting various environments including deep sea waters, temporarily or permanently frozen soils, and even food products. Both ecological groups persist in a state of successional dynamics, with one population outcompeting another as environmental conditions change (Helmke and Weyland 2004).

The biodiversity, ecological role and molecular basis of the adaptation to psychrophilicity of microorganisms inhabiting cold environments have been extensively studied in recent years. The increased interest in cold-adapted bacteria is a consequence of biotechnological application of cold-active enzymes (e.g. in the food and chemical industries), which exhibit high catalytic activity at low temperatures and low thermostability at elevated temperatures (Cavicchioli et al. 2002). Over 40 complete genomic sequences of ‘psychro’ bacteria have been determined so far. These include three strains of the genus *Psychrobacter* (*Psychrobacter arcticus* 273-4, *Psychrobacter cryohalolentis* K5 and *Psychrobacter* sp. PRwf-1)—bacteria frequently isolated from cold environments, but still largely uncharacterized.

*Psychrobacter* spp. comprise a group of gram-negative, rod-shaped, non-motile, aerobic and usually non-pigmented, heterotrophic bacteria, which inhabit Arctic and Antarctic ornithogenic soils, polar permafrost, sea ice and water, as well as living organisms (strains have been isolated from krill and fishes) (e.g. Ayala-del-Río et al. 2010; Bozal et al. 2003). Preliminary information concerning the genome composition of these bacteria has been provided by DNA sequencing projects. However, knowledge of their mobile genetic elements (MGE), i.e. plasmids and transposons, is still very fragmentary.

MGEs play a crucial role in horizontal gene transfer (HGT) and may serve as the basis for the construction of genetic tools useful for biotechnology. Until now, only a few such elements have been identified in *Psychrobacter* spp.: a bacteriophage Psymv2 (Meiring et al. 2012), a composite transposon Tn*5080* (7193 bp; contains streptomycin and tetracycline resistance genes) (Petrova et al. 2009) and four plasmids—plasmid 1 (41221 bp) of *P. cryohalolentis* K5 (composite replicon containing two replication systems), pP62BP1 (34467 bp) of *Psychrobacter* sp. DAB_AL62B, and pRWF101 (13956 bp) and pRWF102 (2117 bp) of *Psychrobacter* sp. PRwf-1. Only pP62BP1 (characterized in our previous study) contains a defined genetic module of adaptive value that has been linked with a specific phenotype, namely transformation of alkyl sulfates into acyl-CoA, with dodecyl sulfate (SDS) as a possible substrate (organic sulfates metabolism) (Lasek et al. 2012). The other three plasmids seem to be cryptic replicons encoding proteins involved in replication, stabilization and conjugal transfer, as well as hypothetical proteins, mostly of unknown function.

In this study we performed an in-depth characterization of a pool of plasmids of six strains of *Psychrobacter* spp. isolated from Spitsbergen island in the Arctic, and we show their relationship to other plasmids that have previously been identified in psychrophilic bacteria.

## Materials and methods

### Bacterial strains, plasmids and culture conditions

The following bacterial strains were used in this study: *E. coli* TG1, *Agrobacterium tumefaciens* LBA288 (Hooykaas et al. 1980), *Paracoccus versutus* UW225 (Bartosik et al. 1993), *Alcaligenes* sp. LM16R (Dziewit et al. 2011a), *Pseudomonas putida* KT2442R (provided by G. Jagura-Burdzy) and six *Psychrobacter* spp. strains (DAB_AL12, DAB_AL25, DAB_AL32B, DAB_AL43B, DAB_AL60, DAB_AL109bw). The strains were grown on lysogeny broth (LB) medium (Sambrook and Russell 2001) at 37 °C (*E. coli*), 30 °C (*A. tumefaciens* LBA288, *P. versutus* UW225, *Alcaligenes* sp. LM16R and *P. putida* KT2442R) or 22 and 4 °C (*Psychrobacter* spp.). Plates of solidified LB medium were prepared by the addition of 1.5 % agar. Where necessary, the medium was supplemented with antibiotics: ampicillin (100 μg/ml), kanamycin (50 μg/ml for strains TG1, UW225, LBA288, KT2442R and *Psychrobacter* spp.; or 200 μg/ml for LM16R) and rifampin (50 μg/ml). The plasmid vectors used in this study were pABW1 (Bartosik et al. 1997), pBGS18 (Spratt et al. 1986), pBluescript SK II (Alting-Mees and Short 1989) and pRK2013 (Ditta et al. 1980).

### Sample collection and bacterial isolation

*Psychrobacter* spp. strains were isolated from guano of little auks (dovekie; *Alle alle*). Guano samples were collected in August 2005 from a breeding colony situated on Spitsbergen island (mountain slope of Ariekammen Isbjornhamna Bay—Hornsund Fjord; 77°00′37.0″N, 15°31′49.5″E). For bacterial isolation, 6 independent samples of guano were taken (1 g wet weight each) and homogenized in 10 ml of 0.85 % NaCl (pH 7.0) in conical Pyrex bottles (100 ml) by mixing vigorously with glass beads (120 rpm for 20 min at 5 °C). After waiting 20 min to allow larger particles to settle, a series of supernatant dilutions were prepared in saline. Aliquots of 100 μl of the respective dilutions were plated on nutrient agar medium and incubated at 4 °C. All operations were carried out aseptically (Zmuda-Baranowska 2010).

### 16S rRNA gene amplification

A colony PCR method was used for the amplification of 16S rRNA gene fragments (Gathogo et al. 2003). PCR was performed with the primers 27f and 1492r (Lane 1991). The amplified 16S rDNA fragments were used as templates for DNA sequencing.

### DNA sequencing

Nucleotide sequences of *Psychrobacter* spp. plasmids and PCR-amplified 16S rDNA fragments were determined using a dye terminator sequencing kit and an automated sequencer (ABI 377 Perkin Elmer) in the DNA Sequencing and Oligonucleotide Synthesis Laboratory at the Institute of Biochemistry and Biophysics, Polish Academy of Sciences. Restriction fragments of *Psychrobacter* plasmids were cloned into compatible sites of vectors pBGS18 and pBluescript SK II for DNA sequencing. Primer walking was used to obtain the full nucleotide sequences of particular plasmids.

### DNA manipulations and introduction of plasmid DNA into bacterial cells

Isolation of plasmid DNA and common DNA manipulation techniques were performed as described by Sambrook and Russell (2001). Transformation of *E. coli* strains was performed according to the method of Kushner (1978). Triparental mating was performed as described previously (Bartosik et al. 2001).

### Temperature and pH tolerance

The temperature and pH tolerance of *Psychrobacter* spp. isolates were measured by following changes in the optical density of cultures (in comparison with non-inoculated controls) grown in titration plates, using an automated microplate reader (Sunrise TECAN). Overnight cultures were diluted in fresh LB medium (pH 7.0 for the temperature tolerance analysis and pH 2.0–13.0 for the pH tolerance analysis) to obtain an initial optical density at 600 nm (OD_600_) of 0.05. Culture aliquots were dispensed into microplates and these were incubated with shaking at 22 °C (for pH tolerance analysis) or 4, 15, 22, 25, 30 or 37 °C (for temperature tolerance analysis) for 72 h.

### Antibiotic, heavy metal and metalloid ion resistance

The minimum inhibitory concentrations (MICs) of ampicillin, chloramphenicol, kanamycin and tetracycline were determined by Epsilometer tests (*E* tests, bioMérieux), with a gradient of the appropriate antibiotic. Each *E* test strip was placed on lawns of bacteria on an agar plate. The pattern of bacterial growth was examined after 48 h of incubation at 22 °C. The lowest concentration of the antibiotic that prevented growth was considered the MIC.

Analytical grade heavy metal salts (3CdSO_4_ × 8H_2_O; CoSO_4_ × 7H_2_O; CuSO_4_; HgCl_2_; K_2_Cr_2_O_7_; NaAsO_2_; Na_2_HAsO_4_ × 7H_2_O; NiCl_2_ × 6H_2_O; ZnSO_4_ × 7H_2_O) were used to prepare 0.01, 0.1 or 1 M stock solutions in water. Each solution was filter-sterilized and added to LB medium to final concentrations of between 0.01 and 100 mM of the metal ion. Exact MICs for the *Psychrobacter* isolates were defined on titration plates using a broth dilution method (Sunrise, TECAN) for measuring changes in the optical density of the cultures in comparison with non-inoculated controls. Each microplate was checked for growth at 24-h intervals for 3 days.

The ability to grow in the presence of (1) 10 mM As(V), (2) 1 mM each of As(III), Cd, Co, Cu, Ni, Zn and Cr, and (3) 0.1 mM Hg was considered a resistance phenotype (Abou-Shanab et al. 2007; Nieto et al. 1987).

### Bioinformatic analysis

Plasmid nucleotide sequences were analyzed using Clone Manager (Sci-Ed8) and Artemis software (Carver et al. 2008). Similarity searches were performed using the BLAST programs (Altschul et al. 1997) provided by the NCBI (http://blast.ncbi.nlm.nih.gov/Blast.cgi) and the PRIAM tool (Claudel-Renard et al. 2003). Helix-turn-helix (HTH) motifs were predicted using the HELIX-TURN-HELIX MOTIF PREDICTION program (Dodd and Egan 1990). Phylogenetic analyses were performed using the Phylogeny Inference Package—PHYLIP v3.69 (Felsenstein 1989), applying the neighbor-joining algorithm with Kimura corrected distances and 1000 bootstrap replicates. Initial alignments obtained with ClustalW (Chenna et al. 2003) were manually refined using the T-Coffee—Multiple Sequence Alignment (Notredame et al. 2000). Trees were rendered with TreeView version 1.6.6 (Page 1996).

### Nucleotide sequence accession numbers

All 16S rDNA sequences determined in this study have been deposited in the GenBank database with the accession numbers JF714889 (DAB_AL12), JF714893 (DAB_AL25), JF714884 (DAB_AL32B), JF714885 (DAB_AL43B), JF714887 (DAB_AL60) and JF714890 (DAB_AL109bw). The nucleotide sequences of plasmids pP12P1, pP32BP1, pP43BP1, pP43BP2, pP43BP3, pP43BP4, pP109bwP1, pP60P1 and pP60P2 have been annotated and deposited in GenBank with respective accession numbers JQ231228, JQ245699, JQ245700, JQ245701, JQ348845, JQ348844, JQ245702, JQ245703 and JQ245704.

## Results

### Isolation and identification of a pool of strains of *Psychrobacter* spp.

Bacterial strains were isolated from guano of little auks—the most abundant Arctic sea birds. A random selection of 88 isolates (Table S1, Supplementary Materials) (obtained from 6 independent guano samples) was examined for their colony and cell morphology, as well as basic physiological features (data not shown). Fragments of 16S rDNA were amplified by PCR from the strains and sequenced. Comparative analysis of the obtained sequences revealed that 17 of the strains (19.3 %) could be classified to the genus *Psychrobacter*. One of the randomly chosen strains (*Psychrobacter* sp. DAB_AL62B) was already described in our previous study (Lasek et al. 2012). The remaining strains were subjected to further analysis. To avoid the characterization of strains of clonal origin, isolates from different guano samples were examined.

**Fig. 1:**
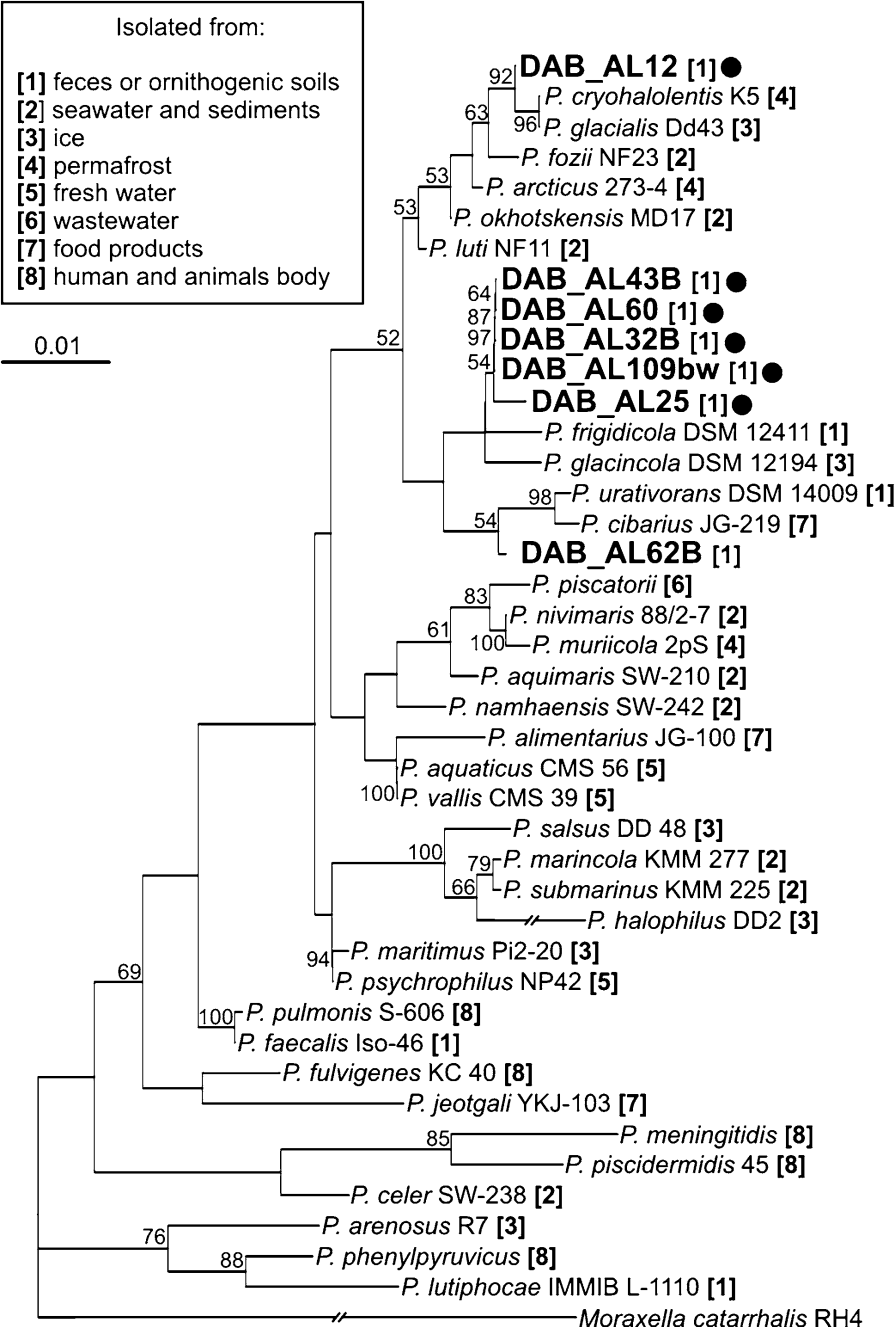
Phylogenetic tree for 16S rDNA sequences of *Psychrobacter* spp. The tree was constructed by the neighbor-joining algorithm with Kimura corrected distances. The statistical support for the internal nodes was determined by 1000 bootstrap replicates and values of >50 % are shown. ‘DAB’ strains are denoted by *bold text* and strains analyzed in this study are indicated by *black dots*. The types of environment from which particular strains were isolated are indicated in *square parenthesis* by the numerical code, explained in the legend next to the tree. The 16S rDNA sequence of *Moraxella catarrhalis* RH4 was used as the outgroup

Phylogenetic analysis was performed, based on the comparison of partial 16S rDNA sequences (1351 bp) of the strains and type strains representing the 34 *Psychrobacter* species described to date. The analyzed sequences of the strains DAB_AL32B, DAB_AL43B, DAB_AL60 and DAB_AL109bw were identical, and share 99.70 % similarity with DAB_AL25. The topology of the phylogenetic tree revealed that these five strains form a separate cluster linked to *P. frigidicola* DSM 12411 (99.11 % identity of the 16S rDNA sequences), isolated from ornithogenic soil in Antarctica (Bowman et al. 1996) (Fig. 1). The strains DAB_AL62B and DAB_AL12 were clustered in different groups. The former is most related to *P. urativorans* DSM 14009 (99.26 % identity) and *P. cibarius* JG-219 (99.18 % identity), isolated from ornithogenic soil and food products, respectively (Bowman et al. 1996; Jung et al. 2005), while the latter (most divergent among the “DAB” strains) is closest to *P. cryohalolentis* K5 (99.78 % identity), isolated from a cryopeg taken from the permafrost in the Kolyma lowland (Siberia, Russia) (Bakermans et al. 2006) (Fig. 1).

### Characterization of the *Psychrobacter* spp. strains

A preliminary characterization of the isolated *Psychrobacter* spp. strains revealed that all of them fulfill the requirements of psychrophilicity (Morita 1975): they could grow at temperatures ranging between 4 and 25 °C, but not at ≥30 °C (optimum temperature 22 °C) (Figure S1, Supplementary Materials). These strains also grew in LB medium at pH values between 5 and 10, which is typical for neutrophilic bacteria (Slonczewski et al. 2009).

Possible resistance phenotypes, which are often determined by plasmids and transposons, were also examined. None of the *Psychrobacter* spp. strains were resistant to any of the tested antibiotics (ampicillin, chloramphenicol, kanamycin and tetracycline), but they showed resistance to several heavy metals: (1) low or moderate resistance to zinc, chromium(VI) and copper (MICs from 2 to 4 mM), and (2) moderate or high level resistance to arsenate—As(V) (MICs from 15 to 100 mM), except DAB_AL25 (MIC 2 mM). Two strains (DAB_AL12 and DAB_AL32B) also exhibited trigger level resistance to arsenite—As(III) (MICs of 1.5 and 2 mM, respectively).

### Identification of a pool of *Psychrobacter* spp. plasmids

Plasmids are natural vectors that play a major role in the dissemination of accessory genetic information in HGT. The *Psychrobacter* strains analyzed in this study were found to carry nine circular plasmids (ranging in size from 2.9 to 14.9 kb) that are listed in Table 1. The highest number of plasmids was found in DAB_AL43B (4) and DAB_AL60 (2), while the remaining strains possess only single replicons or none at all.

**Table 1:** General features of *Psychrobacter* plasmids

Plasmid name	*Psychrobacter* strain	Plasmid size (bp)	GC content (mol%)	Number of ORFs	Average ORF length (bp)	Percentage of coding regions
pP12P1	DAB_AL12	2917	35.7	2	749	51.3
pP32BP1	DAB_AL32B	4599	42.7	6	599	78.1
pP43BP1	DAB_AL43B	4390	37.2	6	593	81.0
pP43BP2	DAB_AL43B	5445	37.3	6	615	67.8
pP43BP3	DAB_AL43B	4955	39.2	7	520	73.5
pP43BP4	DAB_AL43B	6450	41.3	9	705	81.0
pP60P1	DAB_AL60	5435	41.7	8	710	83.7
pP60P2	DAB_AL60	14924	37.8	13	858	74.7
pP109bwP1	DAB_AL109bw	4402	42.9	6	725	74.5

The complete nucleotide sequences of these plasmids were determined. The relatively low average GC content of the obtained sequences (35.7–42.9 mol%) is typical for *Psychrobacter* spp. genomic DNA (e.g. Ayala-del-Río et al. 2010). The plasmids were found to contain from 2 to 13 open reading frames (ORFs), of sizes between 165 and 2133 bp (Table 1). Based on similarities to known genes, it was possible to predict functions for the polypeptides encoded by almost half of these ORFs. A summary of the ORFs, including their position, transcriptional orientation, the size of the encoded proteins, and their closest known homologs, is presented in Table S2 (Supplementary Materials).

**Fig. 2:**
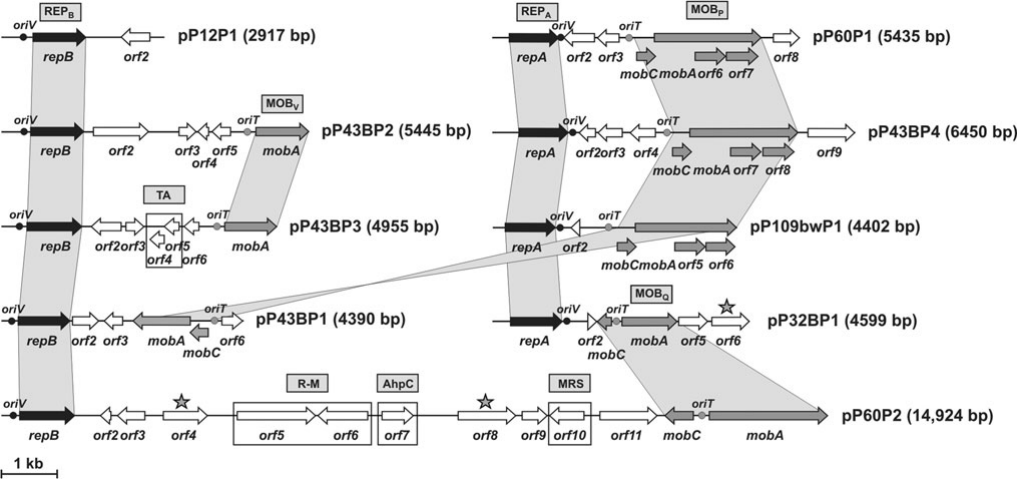
Linear maps showing the genetic structure of circular plasmids pP12P1, pP32BP1, pP43BP1, pP43BP2, pP43BP3, pP43BP4, pP60P1, pP60P2 and pP109bwP1. The predicted genetic modules are indicated by *gray rectangles*: *REP*_*A*_ (REP_B_) *repA*-like (*repB*-like) replication systems, *MOB*_*P*_ (MOB_Q_, MOB_V_) system for mobilization for conjugal transfer, *TA* toxin–antitoxin system, *R-M* type II restriction-modification system, *MRS* multimer resolution system, *AhpC* subunit C of alkyl hydroperoxide reductase module. *Gray stars* indicate Sel1 domain repeat-containing proteins with slight homology to beta-lactam hydrolases. *Arrows* indicate the transcriptional orientation of the genes. Areas of *gray shading* connect genes of different plasmids encoding related proteins

Further analysis of the organization of the plasmids revealed the presence of several putative genetic modules responsible for (1) plasmid replication (REP), (2) stabilization (STA), and (3) mobilization for conjugal transfer (MOB). All plasmids also appear to carry different accessory genetic information (Fig. 2).

### REP modules: structure and host range

Bioinformatic analysis of the plasmid genomes indicated the presence of two types of REP module: (1) *repB*-like (pP12P1, pP43BP1, pP43BP2, pP43BP3 and pP60P2) and (2) *repA*-like (pP32BP1, pP43BP4, pP60P1 and pP109bwP1). The sequences of these modules could be differentiated by their GC content: 35.7–39.2 mol% for *repB*-like and 41.3–42.9 mol% for *repA*-like plasmids (Table 1).

**Fig. 3:**
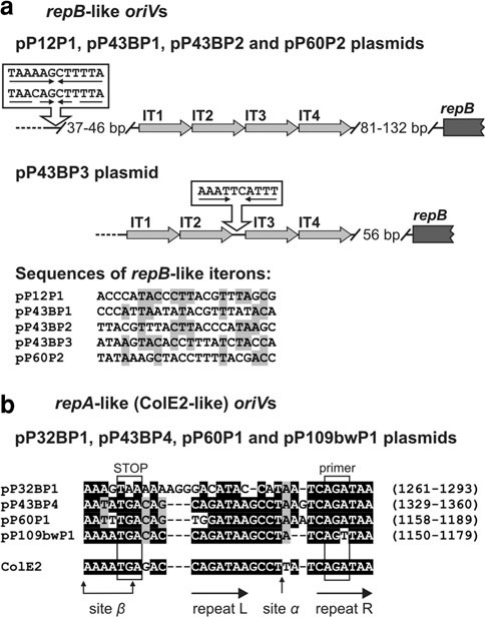
Structure and alignments of *repB*-like *oriV*s and iterons (**a**) and *repA*-like *oriV*s (**b**) of the analyzed *Psychrobacter* plasmids. Nucleotides identical to the reference sequence of *oriV* of ColE2 are shown against a *black background* and those common to at least 50 % of the analyzed sequences have a *gray background*

The characterized REP modules of pP12P1, pP43BP1, pP43BP2, pP43BP3 and pP60P2 have a structure that is typical for the replication systems of many theta-replicating plasmids (Chattoraj 2000). They contain a single ORF encoding a predicted protein with similarities to the initiator RepB protein, possessing nicking-closing (topoisomerase I like) activity, and a putative origin of replication (*oriV*), placed upstream of the *repB* gene (Fig. 3a). Comparative analysis revealed that closely related replication proteins are also encoded by plasmid 1 of *P. cryohalolentis* K5 (acc. no. NC_007968) and many plasmids of *Acinetobacter* spp. strains (e.g. pMAC and p11921 of *Acinetobacter baumannii*, acc. nos. NC_006877 and GU979000, respectively).

The putative *oriV*s of pP12P1, pP43BP1, pP43BP2, p43BP3 and pP60P2 contain four tandemly placed 22-bp long repeated sequences (IT1–IT4), i.e. putative iterons, which (as shown for other related plasmids of this type; Chattoraj 2000) may constitute binding sites for the Rep proteins. The IT repeats are located 81–132 bp upstream of the *repB* genes. They are identical in particular plasmids (with one exception being IT4 of pP43BP2, which differs from IT1 to IT3 by 5 mismatches); however, they show only limited reciprocal sequence similarity (Fig. 3a). Interestingly, the plasmids pP12P1, pP43BP1, pP43BP2 and pP60P2 contain a conserved palindromic sequence [5′-TAA(A/C)AGCTTTTA-3′] located 37–46 bp upstream of the IT1 repeats (Fig. 3a). In the case of pP43BP3, a similar sequence (5′-AAATTCATTT-3′) is situated between the IT2 and IT3 repeats (Fig. 3a). High conservation of the palindromic structure might suggest a role in replication initiation.

The REP modules of the second group of plasmids (pP32BP1, pP43BP4, pP60P1 and pP109bwP1) contain a single ORF encoding a putative protein in which three conserved regions can be distinguished: (1) the replicase domain, typical for plasmid DNA replication initiator proteins, (2) an alpha helical domain, found in the C-terminal regions of primases (PriCT-1), and (3) a HTH motif, that is most probably responsible for protein–DNA interactions (data not shown). The analyzed proteins share significant amino acid sequence homology with the RepA proteins of plasmid 1 of *P. cryohalolentis* K5 and pRWF101 of *Psychrobacter sp.* PRwf-1 (acc. nos. NC_007968 and NC_009516, respectively), and with the replication initiation protein of *E. coli* plasmid ColE2 (Yasueda et al. 1989). Careful inspection of the nucleotide sequences of the analyzed REP modules also revealed the presence of three DNA regions typical for the origins of replication of ColE2-type plasmids: (1) two direct repeats (L and R) 5′-CAGATAA-3′, (2) sites α and β, which determine the specificity of the interactions of Rep protein with the origin, and (3) a short sequence to which the Rep protein synthesizes a unique RNA primer that is crucial for the initiation of leading-strand DNA synthesis by DNA polymerase I (Fig. 3b) (Yagura et al. 2006).

The host range of REP modules representing both of the aforementioned groups of replicons was then examined. For this analysis we used two shuttle plasmids, pABW-12P1 and pABW-60P1, containing the REPs of pP60P1 and pP12P1, respectively, cloned (within *Xba*I restriction fragments) into the *E. coli*-specific, narrow host range (NHR) vector pABW1 (ColE1-type *ori* of pMB1). The ability of the shuttle plasmids to replicate was tested in (1) *A. tumefaciens* LBA 288R and *P. versutus* UW225 (*Alphaproteobacteria*), (2) *Alcaligenes* sp. LM16R (*Betaproteobacteria*), and (3) *P. putida* KT2442R (*Gammaproteobacteria*). The plasmids pABW-12P1 and pABW-60P1 were found to replicate exclusively in *Psychrobacter* spp., suggesting a relatively NHR.

### Stable maintenance modules

Most of the analyzed plasmids appeared to lack stabilization systems, which are components of the vast majority of bacterial replicons. Only plasmid pP43BP3 carries a complete toxin–antitoxin (TA) system, possibly involved in the postsegregational elimination of plasmid-less cells from a bacterial population (Fig. 2). This putative TA module is composed of two short overlapping ORFs (14 bp overlap) encoding proteins with similarity to a number of RelB-like antitoxins (ORF5) and RelE-like toxins (ORF4) of *relBE*/*parDE*-type TA systems (Anantharaman and Aravind 2003). Two other plasmids, pP43BP2 and pP109bwP1, carry incomplete TA modules represented by single ORFs encoding RelB-like antitoxins.

Another plasmid, pP60P2, contains a putative type II restriction-modification (R-M) system. Similar to TA, such systems may increase the stability of plasmids by killing plasmid-less cells (e.g. Ichige and Kobayashi 2005; Dziewit et al. 2011b). The RM module of pP60P2 is composed of two divergently orientated ORFs: ORF5 and ORF6 (Fig. 2). BLAST searches revealed that the polypeptide encoded by ORF5 shares substantial homology with a large number of proteins annotated as m5C methyltransferases (MTases), with highest sequence similarity (60 %) to a putative MTase of *Marivirga tractuosa* DSM 4126 (acc. no. YP_004054233). The ORF6-encoded protein is similar to a putative restriction endonuclease (predicted recognition sequence 5′-CGCG-3′) encoded by *Moraxella catarrhalis* BC1 (acc. no. ZP_11632318).

ORF10 of pP60P2 (Fig. 2) encodes a predicted protein with a catalytic domain characteristic of serine recombinases, often recognized as resolvases in multimer resolution systems (MRS). MRS act to resolve plasmid oligomers and this activity increases the number of independent plasmid molecules available for distribution during cell division (Bahl et al. 2009). Homologs of ORF10 have been identified in other *Psychrobacter* plasmids, including pRWF101 of PRwf-1 and pP62BP1 of DAB_AL62B (Lasek et al. 2012).

### MOB modules: structure and diversity

Many bacterial plasmids can be mobilized for conjugation by other self-transmissible elements (e.g. conjugative plasmids and integrative and conjugative elements—ICEs) encoding type 4 secretion systems. The mobilizable plasmids contain MOB modules encoding specific relaxosome components and an origin of transfer (*oriT*) (Francia et al. 2004; Garcillan-Barcia et al. 2009). The analyzed plasmids of *Psychrobacter* spp. were found to contain eight putative MOB modules, which may be classified, based on amino acid sequence similarities of their relaxases, into three distinct families (MOB_P_, MOB_Q_ and MOB_V_).

**Fig. 4:**
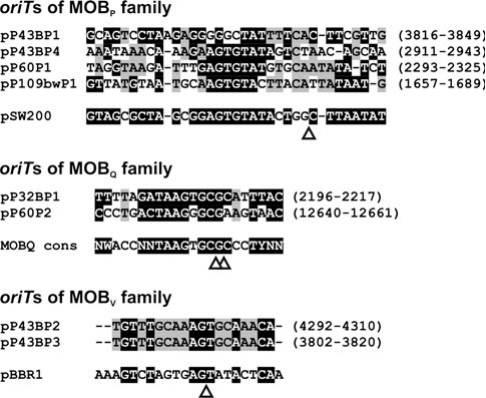
Structure and alignments of *oriT*s of the analyzed *Psychrobacter* plasmids. Nucleotides identical to the reference (*oriT* of pSW200 and *oriT* of pBBR1) or consensus sequences (*oriT* of MOB_Q_ modules) are shown against a *black background* and those common to at least 50 % of the analyzed sequences have a *gray background*. The positions of the predicted *oriT*s within the sequences of the plasmids are shown in *parenthesis*. Experimentally determined or predicted (for pSW200) *nic*-cleavage sites are indicated with a *triangle*

Analysis of the MOB_P_ family [MOB_P5_ (MOB_HEN_) clade] members, found in plasmids pP43BP1, pP43BP4, pP60P1 and pP109bwP1, revealed their structural divergence and permitted the identification of two subgroups. The single member of the first subgroup (MOB of pP43BP1) is composed of two overlapping ORFs (putative *mobA* and *mobC*), while the other three MOB_P_ modules carry four convergently orientated ORFs (Fig. 4). The MobA relaxases of the two subgroups show partial amino acid sequence identity (≤36 %). The pP43BP1 MobA is most similar (44 % identity) to the relaxase/mobilization nuclease domain protein of plasmid pRWF101 of *Psychrobacter* sp. PRwf-1 (acc. no. NC_009516). In turn, the MobA proteins of pP43BP4, pP60P1 and pP109bwP1 are most similar (≥57 % identity) to the relaxase encoded by plasmid pKW1 of *Pseudoalteromonas* sp. 643A (Cieśliński et al. 2008). Interestingly, the three relaxases of this second subgroup are at least twice the length of other MobA proteins, and their C-terminal parts show no similarity to known protein sequences present in the GenBank (NCBI) databases. All of the analyzed MOB_HEN_ modules contain predicted *oriT*s (located upstream of the *mobC* genes), which share sequence similarity with *oriT* of the mobilization system (MOB_HEN_ clade member) of plasmid pSW200 of *Erwinia stewartii* SW2 (Fu et al. 1998) (Fig. 4).

The MOB_Q_ family is the most abundant and diverse group of mobilization systems. Two such modules (encoding relaxases most similar to proteins classified within the MOB_Q3_ clade; Garcillan-Barcia et al. 2009) were identified within plasmids pP32BP1 and pP60P2. Both MOBs are composed of two non-overlapping, divergently orientated ORFs, encoding a putative relaxase (MobA) and a mobilization protein C (MobC), as well as a predicted *oriT* located within the *mobA*–*mobC* intergenic region (Fig. 4). The *oriT*s display 55 % sequence identity and they match the *oriT* consensus sequence of the MOB_Q_ family (Francia et al. 2004) (Fig. 4). The relaxases of pP32BP1 and pP60P2 differ significantly in length and they exhibit only a moderate level of sequence similarity (40 %). Similarity searches revealed that the predicted proteins are most closely related (52 %) to the MobA protein encoded by a small (4658 bp) cryptic plasmid pMbo4.6 of *Moraxella bovis* ATCC 10900 (acc. no. NC_013500).

The MOB modules of plasmids pP43BP2 and pP43BP3 (Fig. 4) encode related relaxases (85 % identity), which exhibit ≥40 % amino acid sequence homology with the Mob protein of the mobilization system of a broad host range plasmid pBBR1 (2687 bp) of *Bordetella bronchiseptica* S87—the prototype of the MOB_V2_ clade (MOB_V_ family) (Antoine and Locht 1992; Garcillan-Barcia et al. 2009). The predicted *oriT*s of these plasmids (both placed upstream of the *mobA* genes) are identical, and differ slightly from the *oriT* of pBBR1 (Fig. 4). They contain 8-bp long inverted repeats (IR) separated by a 3-bp spacer, which is a typical structure for *oriT*s of MOBs of the MOB_V_ family (Szpirer et al. 2001; Guzman and Espinosa 1997).

### Accessory genetic information

Besides the REP, MOB and STA modules, the analyzed plasmids were found to contain accessory genetic information (33 ORFs in total) of unknown function. Using the PRIAM program for automated enzyme detection we were able to assign putative functions to only four of the identified ORFs. ORF4 and ORF8 of pP60P2 and ORF6 of pP32BP1 (Fig. 2) encode putative Sel1 domain proteins, with weak homology (35–39 % amino acid sequence similarity; *E* value >1e−10) to beta-lactam hydrolases (EC 3.5.2.6)—a group of enzymes of varying specificity that hydrolyze penicillins or cephalosporins. ORF7 of plasmid pP60P2 encodes a putative protein with 99 % amino acid sequence similarity to subunit C of alkyl hydroperoxide reductase (EC 1.11.1.15) encoded by the *ahpC* gene of *Psychrobacter arcticus* 273-4. This enzyme reduces H_2_O_2_, organic peroxides and peroxynitrite (ONOO^−^), and therefore acts as an antioxidant and major scavenger of reactive oxygen species (ROS) generated in the cytoplasm of bacteria as a by-product of aerobic metabolism (Chen et al. 1998; Steele et al. 2010).

## Discussion

In this study we have performed a detailed analysis of the plasmidome of six Arctic strains of *Psychrobacter* spp. The effect of temperature and pH on the growth of these strains in culture was similar, which permitted their classification as neutrophilic psychrophiles. The strains also showed a low or moderate level of resistance against arsenic(III), zinc, chromium(VI) and copper, and a high level of tolerance to arsenic(V). Since planktivorous seabirds are efficient vectors for the transport of heavy metals between marine and terrestrial ecosystems (e.g. Yin et al. 2008), these resistance phenotypes may be a consequence of the constant exposure of bacteria to heavy metals accumulated in little auk feces. Nevertheless, none of the plasmids analyzed in this study contain heavy metal resistance determinants.

The isolated *Psychrobacter* spp. strains carried nine plasmids in total, containing either a *repA*-like (pP32BP1, pP43BP4, pP60P1 and pP109bwP1) or *repB*-like (pP12P1, pP43BP1, pP43BP2, pP43BP3 and pP60P2) replication system. REP modules are major components of plasmid backbones, and therefore they serve as exclusive phylogenetic markers for classification of these replicons (e.g. Petersen et al. 2009). We performed in-depth searches of the NCBI databases in order to define all known plasmids of psychrophilic bacteria. This analysis revealed the presence of only 45 plasmids (including the 9 replicons analyzed in this study) identified in 27 bacterial strains (the vast majority of the plasmid sequences were derived from whole genome sequencing projects).

**Fig. 5:**
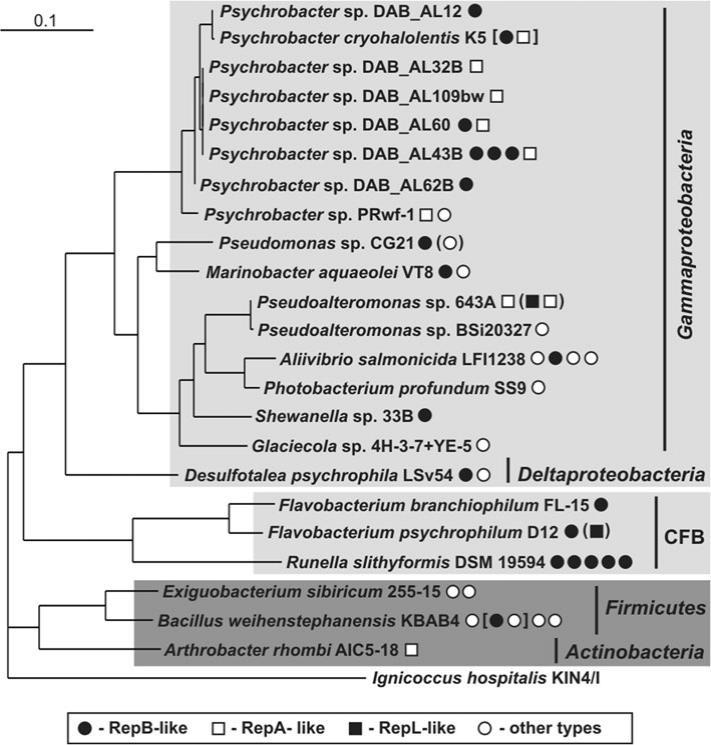
Phylogenetic tree for 16S rDNA sequences of psychrophilic bacteria harboring plasmids. The tree was constructed by the neighbor-joining algorithm with Kimura corrected distances, and the statistical support for the internal nodes was determined by 1000 bootstrap replicates. The 16S rDNA sequence of a representative of the *Archaea*, *Ignicoccus hospitalis KIN4/I*, was used as the outgroup. Gram-negative and gram-positive bacteria are shown against *dark and light gray backgrounds*, respectively. The type of replication system of each analyzed plasmid (based on sequence alignments, which are summarized in the Supplementary Materials, Figs. S2, S3 and S4) is indicated by a *dot or square*: *black dot* RepB-like, *white square* RepA-like, *black square* RepL-like, *white dot* other types. *Dots within square* *parenthesis* represent replication systems of composite replicons, and *dots within round parenthesis* represent replication modules of plasmids of *Pseudomonas* sp. MC1, *Pseudoalteromonas* spp. (Bsi429, PS1M2 and PS1M3) and *Flavobacterium* sp. KP1 (whose 16S rDNA sequences were not available in the GenBank database). Accession numbers of all plasmids whose replication proteins were used for the analysis are given in the Supplementary Materials, Table S3

Most of the analyzed plasmids of psychrophilic bacteria (20 replicons; 44.4 %) contain RepB-like replication systems. Such replicons have been identified in bacteria representing distinct phylogenetic groups including (1) gram-negative [*Gammaproteobacteria* (*Marinobacter* sp., *Pseudomonas* sp., *Shewanella* sp., *Psychrobacter* spp.), *Deltaproteobacteria* (*Desulfotalea psychrophila* LSv54), and *Cytophaga*–*Flavobacterium*–*Bacteroides* (CFB) group (*Flavobacterium* spp., *Runella slithyformis* DSM 19594)] and (2) gram-positive hosts (*Bacillus weihenstephanensis* KBAB4) (Fig. 5). Interestingly, some strains contain more than one RepB-like plasmid, e.g. *Psychrobacter* sp. DAB_AL43B (analyzed in this study) carries 3 such plasmids, and *Runella slithyformis* DSM 19594 has 5 such plasmids (Copeland et al. 2012) (Fig. 5). Despite the presence of closely related REP modules, these plasmids belong to various incompatibility groups, thus obeying the rule that coexisting replication systems have to be compatible (Petersen et al. 2009). RepB-like modules have also been identified within two composite replicons: plasmid 1 of *P. cryohalolentis* K5 (acc. no. NC_007968) and pBWB402 of *B. weihenstephanensis* KBAB4 (Rasko et al. 2005). Interestingly, the former plasmid contains both RepB-like and RepA-like (ColE2-type) replication systems.

RepA-like modules are also frequently found (9 replicons; 20 %) within plasmids of psychrophilic *Gammaproteobacteria* (*Pseudoalteromonas* spp., *Psychrobacter* spp.) and *Actinobacteria* (*Arthrobacter rhombi*) (Fig. 5). The remaining plasmids of psychrophilic bacteria contain (1) REP modules of the Firmicute RepL type (identified in *Pseudoalteromonas* spp. and *Flavobacterium psychrophilum* D12) (3 replicons; 6.7 %), and (ii) single REPs that are unique to psychrophiles (16 replicons; 35.5 %) (Fig. 5).

All plasmids of *Psychrobacter* spp. analyzed in this study have a relatively NHR, limited to this genus. Eight contain MOB systems (classified within the MOB_Q_, MOB_V_ or MOB_P_ families), which suggests the possibility of their mobilization by conjugal transfer. As shown by Smorawinska et al. (2012) such NHR, mobilizable plasmids are efficient carrier molecules, since they may act as natural suicide vectors, promoting the spread of diverse genetic information among evolutionarily distinct bacterial species.

Each of the analyzed plasmids also carries an additional genetic load. Most of the predicted ORFs encode hypothetical proteins of unknown function (this is also the case for *Psychrobacter* spp. genomes, which are largely uncharacterized and require further investigation). Two of the analyzed plasmids (pP32BP1 and pP60P2) carry ORFs encoding proteins with Sel1 domains (present in beta-lactam hydrolases), but the host strains of these plasmids did not show resistance to beta-lactam antibiotics. Nevertheless, this is an interesting observation, since the beta-lactamase-like proteins, possibly involved in some aspect of “normal” bacterial metabolism, may in fact represent ancestors of the antibiotic resistance determinants commonly found in environmental strains and clinical isolates (Petrova et al. 2009).

One of the analyzed plasmids (pP60P2) encodes a putative type II restriction-modification system. The main role of R-M systems is to protect the host cell against invasion by exogenous (not protected by methylation) DNA, e.g. bacteriophage genomes (Tock and Dryden 2005). The first bacteriophage able to infect *Psychrobacter* spp. cells was recently described (Meiring et al. 2012). On the other hand, R-M systems may facilitate DNA recombination by the generation of highly recombinogenic, double-stranded breaks in homologous donor DNA, which can significantly enhance bacterial diversity. R-M systems are frequently carried by plasmids, e.g. pP62BP2 of *Psychrobacter* DAB_AL62B contains two nearly identical modules of this type (Lasek et al. 2012). It has been demonstrated that R-M systems act in an analogous manner to TA modules, which stabilize plasmids by eliminating plasmid-less cells from the bacterial population (Kobayashi 2001).

In close proximity to the R-M module, plasmid pP60P2 also contains a single ORF encoding a putative subunit C of alkyl hydroperoxide reductase (AhpC). The AhpC proteins are components of alkyl hydroperoxide reductase complexes—AhpCD or AhpCF. Peroxiredoxins of the AhpC family reduce, and therefore, detoxify H_2_O_2_, organic peroxides and peroxynitrite (ONOO^−^), while AhpD and AhpF are peroxiredoxin reductases that restore the enzymatic activity of AhpC. AhpCD and AhpCF complexes serve as important antioxidants, and the AhpC protein is the major scavenger of ROS produced by aerobic metabolism (Chen et al. 1998; Steele et al. 2010). In a few cases it has been demonstrated that AhpC proteins may influence the virulence of bacterial pathogens, including *Helicobacter pylori* (Olczak et al. 2003) and *Mycobacterium bovis* (Wilson et al. 1998). Interestingly, it was also shown that mycobacterial AphC could protect human cells against necrosis and apoptosis caused by reactive nitrogen intermediates (RNI) (Chen et al. 1998).

The role of the AhpC proteins in psychrophilic strains may be strictly linked to the environmental conditions they face. Springtime polar ozone depletion and global warming mean that the impact of UV radiation (UVR) remains relatively high in polar regions. This phenomenon, together with other stress-inducing factors (e.g. pollutants), strongly enhances the formation of ROS (Müller et al. 2012; Regoli et al. 2000). Therefore, the acquisition of an *ahpC*-encoding plasmid may potentially increase the adaptation of the host strain to the arctic environment. Further study is required to experimentally confirm this speculation.

In conclusion, the findings of this study greatly increase current knowledge of the mobile DNA of *Psychrobacter* spp. This extended comparative analysis of plasmids has shed light on the distribution of related replicons among psychrophilic bacteria, which, in many cases, reflects the frequency and direction of HGT events. Our results also identify the most ubiquitous “cold-active” plasmid-encoded REP modules, which may form the basis of novel shuttle vectors, specific for this group of bacteria.

## Electronic supplementary material

Below is the link to the electronic supplementary material.

Supplementary Figure S1 (TIFF 14570 kb)

Alignment of RepA-like replication proteins of the following plasmids: pPBS of Pseudoalteromonas sp. Bsi429; pKW1 of Pseudoalteromonas sp. 643A; pP43BP4 of Psychrobacter sp. DAB_AL43B; plasmid 1 of Psychrobacter cryohalolentis K5; pP60P1 of Psychrobacter sp. DAB_AL60; pP32BP2 of Psychrobacater sp. DAB_AL32B; pRWF101 of Psychrobacter sp. PRwf-1; pP109bwP1 of Psychrobacter sp. DAB_AL109bw; pPRH of Arthrobacter rhombi; with ColE2 of E. coli as a reference plasmid. For the accession numbers of particular plasmids, see Table S2 (Supplementary Materials). Identical (within all analyzed sequences) amino acids are shown against a black background and those common to at least 50% of the analyzed sequences have a gray background (TIFF 8501 kb)

Alignment of RepB-like replication proteins of the following plasmids: pBWB402 of Bacillus weihenstephanensis KBAB4; plasmid small of Desulfotalea psychrophila LSv54; pRUNSL01, pRUNSL02, pRUNSL03, pRUNSL04 and pRUNSL05 of Runella slithyformis DSM 19594; pMAQU01 of Marinobacter aquaeolei VT8; pFB1 of Flavobacterium branchiophilum FL-15; pP62BP1 of Psychrobacter sp. DAB_AL62B; pP43BP1, pP43BP2 and pP43BP3 of Psychrobacter sp. DAB_AL43B; pP60P2 of Psychrobacter sp. DAB_AL60; pP12P1 of Psychrobacter sp. DAB_AL12; plasmid 1 of Psychrobacter cryohalolentis K5; pCP1 of Flavobacterium psychrophilum D12; pMWHK01 of Pseudomonas sp. CG21; pVSAL43 of Allivibrio salmonicida LFI1238; and pSFKW33 of Shewanella sp. 33B. For the accession numbers of particular plasmids, see Table S2 (Supplementary Materials). Amino acids common to at least 50% of the analyzed sequences have a gray background (TIFF 14321 kb)

Alignment of RepL-like replication proteins of the following plasmids: pPS1M3 of Pseudoalteromonas sp. PS1M3 and pFL1 of Flavobacterium sp. KP1. For accession numbers of particular plasmids, see Table S2 (Supplementary Materials). Identical (within both analyzed sequences) amino acids are shown against a black background (TIFF 1157 kb)

Supplementary Table S1 (DOC 40 kb)

Supplementary Table S2 (DOC 209 kb)

Supplementary Table S3 (DOC 50 kb)

## References

[CR1] Abou-Shanab RA, van Berkum P, Angle JS (2007). Heavy metal resistance and genotypic analysis of metal resistance genes in gram-positive and gram-negative bacteria present in Ni-rich serpentine soil and in the rhizosphere of *Alyssum murale*. Chemosphere.

[CR2] Alting-Mees MA, Short JM (1989). pBluescript II: gene mapping vectors. Nucleic Acids Res.

[CR3] Altschul SF, Madden TL, Schaffer AA, Zhang J, Zhang Z, Miller W, Lipman DJ (1997). Gapped BLAST and PSI-BLAST: a new generation of protein database search programs. Nucleic Acids Res.

[CR4] Anantharaman V, Aravind L (2003). New connections in the prokaryotic toxin–antitoxin network: relationship with the eukaryotic nonsense-mediated RNA decay system. Genome Biol.

[CR5] Antoine R, Locht C (1992). Isolation and molecular characterization of a novel broad-host-range plasmid from Bordetella bronchiseptica with sequence similarities to plasmids from gram-positive organisms. Mol Microbiol.

[CR6] Ayala-del-Río HL, Chain PS, Grzymski JJ, Ponder MA, Ivanova N, Bergholz PW, Di Bartolo G, Hauser L, Land M, Bakermans C, Rodrigues D, Klappenbach J, Zarka D, Larimer F, Richardson P, Murray A, Thomashow M, Tiedje JM (2010). The genome sequence of *Psychrobacter arcticus* 273-4, a psychroactive Siberian permafrost bacterium, reveals mechanisms for adaptation to low-temperature growth. Appl Environ Microbiol.

[CR7] Bahl MI, Hansen LH, Sørensen SJ (2009). Persistence mechanisms of conjugative plasmids. Methods Mol Biol.

[CR8] Bakermans C, Ayala-del-Río HL, Ponder MA, Vishnivetskaya T, Gilichinsky D, Thomashow MF, Tiedje JM (2006). *Psychrobacter cryohalolentis* sp. nov. and *Psychrobacter arcticus* sp. nov., isolated from Siberian permafrost. Int J Syst Evol Microbiol.

[CR9] Bartosik D, Baj J, Plasota M, Piechucka E, Wlodarczyk M (1993). Analysis of *Thiobacillus versutus* pTAV1 plasmid functions. Acta Microbiol Pol.

[CR10] Bartosik D, Bialkowska A, Baj J, Wlodarczyk M (1997). Construction of mobilizable cloning vectors derived from pBGS18 and their application for analysis of replicator region of a pTAV202 mini-derivative of *Paracoccus versutus* pTAV1 plasmid. Acta Microbiol Pol.

[CR11] Bartosik D, Szymanik M, Wysocka E (2001). Identification of the partitioning site within the *repABC*-type replicon of the composite *Paracoccus versutus* plasmid pTAV1. J Bacteriol.

[CR12] Bowman JP, Cavanagh J, Austin JJ, Sanderson K (1996). Novel *Psychrobacter* species from Antarctic ornithogenic soils. Int J Syst Bacteriol.

[CR13] Bozal N, Montes MJ, Tudela E, Guinea J (2003). Characterization of several *Psychrobacter* strains isolated from Antarctic environments and description of *Psychrobacter luti* sp. nov. and *Psychrobacter fozii* sp. nov. Int J Syst Evol Microbiol.

[CR14] Carver T, Berriman M, Tivey A, Patel C, Böhme U, Barrell BG, Parkhill J, Rajandream MA (2008). Artemis and ACT: viewing, annotating and comparing sequences stored in a relational database. Bioinformatics.

[CR15] Cavicchioli R, Siddiqui KS, Andrews D, Sowers KR (2002). Low temperature extremophiles and their applications. Curr Opin Biotechnol.

[CR16] Chattoraj DK (2000). Control of plasmid DNA replication by iterons: no longer paradoxical. Mol Microbiol.

[CR17] Chen L, Xie QW, Nathan C (1998). Alkyl hydroperoxide reductase subunit C (AhpC) protects bacterial and human cells against reactive nitrogen intermediates. Mol Cell.

[CR18] Chenna R, Sugawara H, Koike T, Lopez R, Gibson TJ, Higgins DG, Thompson JD (2003). Multiple sequence alignment with the Clustal series of programs. Nucleic Acids Res.

[CR19] Cieśliński H, Werbowy K, Kur J, Turkiewicz M (2008). Molecular characterization of a cryptic plasmid from the psychrotrophic antarctic bacterium *Pseudoalteromonas* sp. 643A. Plasmid.

[CR20] Claudel-Renard C, Chevalet C, Faraut T, Kahn D (2003). Enzyme specific profiles for genome annotation: PRIAM. Nucleic Acids Res.

[CR21] Copeland A, Zhang X, Misra M, Lapidus A, Nolan M, Lucas S, Deshpande S, Cheng JF, Tapia R, Goodwin LA, Pitluck S, Liolios K, Pagani I, Ivanova N, Mikhailova N, Pati A, Chen A, Palaniappan K, Land M, Hauser L, Pan C, Jeffries CD, Detter JC, Brambilla EM, Rohde M, Djao OD, Göker M, Sikorski J, Tindall BJ, Woyke T, Bristow J, Eisen JA, Markowitz V, Hugenholtz P, Kyrpides NC, Klenk HP, Mavromatis K (2012). Complete genome sequence of the aquatic bacterium *Runella slithyformis* type strain (LSU 4(T)). Stand Genomic Sci.

[CR22] Ditta G, Stanfield S, Corbin D, Helinski DR (1980). Broad host range DNA cloning system for gram-negative bacteria: construction of a gene bank of *Rhizobium meliloti*. Proc Natl Acad Sci USA.

[CR23] Dodd IB, Egan JB (1990). Improved detection of helix-turn-helix DNA-binding motifs in protein sequences. Nucleic Acids Res.

[CR1000] Dziewit L, Adamczuk M, Szuplewska M, Bartosik D (2011a) DIY series of genetic cassettes useful in construction of versatile vectors specific for *Alphaproteobacteria*. J Microbiol Methods 86:166–174 10.1016/j.mimet.2011.04.016 21569803

[CR24] Dziewit L, Kuczkowska K, Adamczuk M, Radlinska M, Bartosik D (2011). Functional characterization of the type II *Pam*I restriction-modification system derived from plasmid pAMI7 of *Paracoccus aminophilus* JCM 7686. FEMS Microbiol Lett.

[CR25] Felsenstein J (1989). Mathematics vs. evolution: mathematical evolutionary theory. Science.

[CR26] Francia MV, Varsaki A, Garcillán-Barcia MP, Latorre A, Drainas C, de la Cruz F (2004). A classification scheme for mobilization regions of bacterial plasmids. FEMS Microbiol Rev.

[CR27] Fu JF, Hu JM, Chang YS, Liu ST (1998). Isolation and characterization of plasmid pSW200 from *Erwinia stewartii*. Plasmid.

[CR28] Garcillan-Barcia MP, Francia MV, de la Cruz F (2009). The diversity of conjugative relaxases and its application in plasmid classification. FEMS Microbiol Rev.

[CR29] Gathogo EW, Waugh AC, Peri N, Redpath MB, Long PF (2003). Colony PCR amplification of actinomycete DNA. J Antibiot.

[CR30] Guzman LM, Espinosa M (1997). The mobilization protein, MobM, of the streptococcal plasmid pMV158 specifically cleaves supercoiled DNA at the plasmid *oriT*. J Mol Biol.

[CR31] Helmke E, Weyland H (2004). Psychrophilic versus psychrotolerant bacteria—occurrence and significance in polar and temperate marine habitats. Cell Mol Biol (Noisy-le-Grand).

[CR32] Hooykaas PJJ, den Dulk-Ras H, Schilperoort RA (1980). Molecular mechanism of Ti plasmid mobilization by R plasmids: isolation of Ti plasmids with transposon insertions in *Agrobacterium tumefaciens*. Plasmid.

[CR33] Ichige A, Kobayashi I (2005). Stability of *Eco*RI restriction-modification enzymes in vivo differentiates the *Eco*RI restriction-modification system from other postsegregational cell killing systems. J Bacteriol.

[CR34] Jung SY, Lee MH, Oh TK, Park YH, Yoon JH (2005). *Psychrobacter cibarius* sp. nov., isolated from jeotgal, a traditional Korean fermented seafood. Int J Syst Evol Microbiol.

[CR35] Kobayashi I (2001). Behavior of restriction-modification systems as selfish mobile elements and their impact on genome evolution. Nucleic Acids Res.

[CR36] Kushner SR, Boyer HB, Nicosia S (1978). An improved method for transformation of *E. coli* with ColE1 derived plasmids. Genetic engineering.

[CR37] Lane DJ, Stackebrandt E, Goodfellow M (1991). 16S/23S rRNA sequencing. Nucleic acid techniques in bacterial systematics.

[CR38] Lasek R, Dziewit L, Bartosik D (2012). Plasmid pP62BP1 isolated from an Arctic *Psychrobacter* sp. strain carries two highly homologous type II restriction-modification systems and a putative organic sulfate metabolism operon. Extremophiles.

[CR39] Meiring TL, Tuffin IM, Cary C, Cowan DA (2012). Genome sequence of temperate bacteriophage Psymv2 from Antarctic Dry Valley soil isolate *Psychrobacter* sp. MV2. Extremophiles.

[CR40] Morita RY (1975). Psychrophilic bacteria. Bacteriol Rev.

[CR41] Müller R, Desel C, Steinhoff FS, Wiencke C, Bischof K (2012). UV-radiation and elevated temperatures induce formation of reactive oxygen species in gametophytes of cold-temperate/Arctic kelps (*Laminariales*, *Phaeophyceae*). Phycol Res.

[CR42] Nieto JJ, Ventosa A, Ruiz-Berraquero F (1987). Susceptibility of halobacteria to heavy metals. Appl Environ Microbiol.

[CR43] Notredame C, Higgins DG, Heringa J (2000). T-Coffee: a novel method for fast and accurate multiple sequence alignment. J Mol Biol.

[CR44] Nowosielski L (2004). Klimat Spitsbergenu. Gazeta Obserwatora IMGW.

[CR45] Olczak AA, Seyler RW, Olson JW, Maier RJ (2003). Association of *Helicobacter pylori* antioxidant activities with host colonization proficiency. Infect Immun.

[CR46] Page RDM (1996). Tree View: an application to display phylogenetic trees on personal computers. Comput Appl Biosci.

[CR47] Petersen J, Brinkmann H, Pradella S (2009). Diversity and evolution of *repABC* type plasmids in *Rhodobacterales*. Environ Microbiol.

[CR48] Petrova M, Gorlenko Z, Mindlin S (2009). Molecular structure and translocation of a multiple antibiotic resistance region of a *Psychrobacter psychrophilus* permafrost strain. FEMS Microbiol Lett.

[CR49] Przybylak R, Araźny A (2006). Climatic conditions of the north-western part of Oscar II Land (Spitsbergen) in the period between 1975 and 2000. Pol Polar Res.

[CR50] Rasko DA, Altherr MR, Han CS, Ravel J (2005). Genomics of the *Bacillus cereus* group of organisms. FEMS Microbiol Rev.

[CR51] Regoli F, Nigro M, Bompadre S, Winston GW (2000). Total oxidant scavenging capacity (TOSC) of microsomal and cytosolic fractions from Antarctic, Arctic and Mediterranean scallops: differentiation between three potent oxidants. Aquat Toxicol.

[CR52] Sambrook J, Russell DW (2001). Molecular cloning: a laboratory manual.

[CR53] Slonczewski JL, Fujisawa M, Dopson M, Krulwich TA (2009). Cytoplasmic pH measurement and homeostasis in bacteria and archaea. Adv Microb Physiol.

[CR54] Smorawinska M, Szuplewska M, Zaleski P, Wawrzyniak P, Maj A, Plucienniczak A, Bartosik D (2012). Mobilizable narrow host range plasmids as natural suicide vectors enabling horizontal gene transfer among distantly related bacterial species. FEMS Microbiol Lett.

[CR55] Spratt BG, Hedge PJ, te Heesen S, Edelman A, Broome-Smith JK (1986). Kanamycin-resistant vectors that are analogues of plasmids pUC8, pUC9, pEMBL8 and pEMBL9. Gene.

[CR56] Steele KH, Baumgartner JE, Valderas MW, Roop RM (2010). Comparative study of the roles of AhpC and KatE as respiratory antioxidants in *Brucella abortus* 2308. J Bacteriol.

[CR57] Szpirer CY, Faelen M, Couturier M (2001). Mobilization function of the pBHR1 plasmid, a derivative of the broad-host-range plasmid pBBR1. J Bacteriol.

[CR58] Thomas DN, Fogg GE, Convey P, Fritsen CH, Gili J-M, Gradinger R, Laybourn-Parry J, Reid K, Walton DWH (2008). The biology of polar regions.

[CR59] Tock MR, Dryden DT (2005). The biology of restriction and anti-restriction. Curr Opin Microbiol.

[CR60] Wilson T, de Lisle GW, Marcinkeviciene JA, Blanchard JS, Collins DM (1998). Antisense RNA to *ahpC*, an oxidative stress defence gene involved in isoniazid resistance, indicates that AhpC of *Mycobacterium bovis* has virulence properties. Microbiology.

[CR61] Yagura M, Nishio SY, Kurozumi H, Wang CF, Itoh T (2006). Anatomy of the replication origin of plasmid ColE2-P9. J Bacteriol.

[CR62] Yasueda H, Horii T, Itoh T (1989). Structural and functional organization of ColE2 and ColE3 replicons. Mol Gen Genet.

[CR63] Yin X, Xia L, Sun L, Luo H, Wang Y (2008). Animal excrement: a potential biomonitor of heavy metal contamination in the marine environment. Sci Total Environ.

[CR64] Zmuda-Baranowska MJ (2010) Różnorodność bakterii biorących udział w degradacji materii organicznej pochodzenia morskiego w przybrzeżnych środowiskach lądowych Antarktyki i Arktyki. PhD thesis, Polish Academy of Sciences, Warsaw

