# Supplementary figures and images for: Plasmid diversity in arctic strains of Psychrobacter spp
Source: Extremophiles. 2013 Mar 12;17(3):433–44. doi: 10.1007/s00792-013-0521-0 (PMC3632715; doi:10.1007/s00792-013-0521-0)

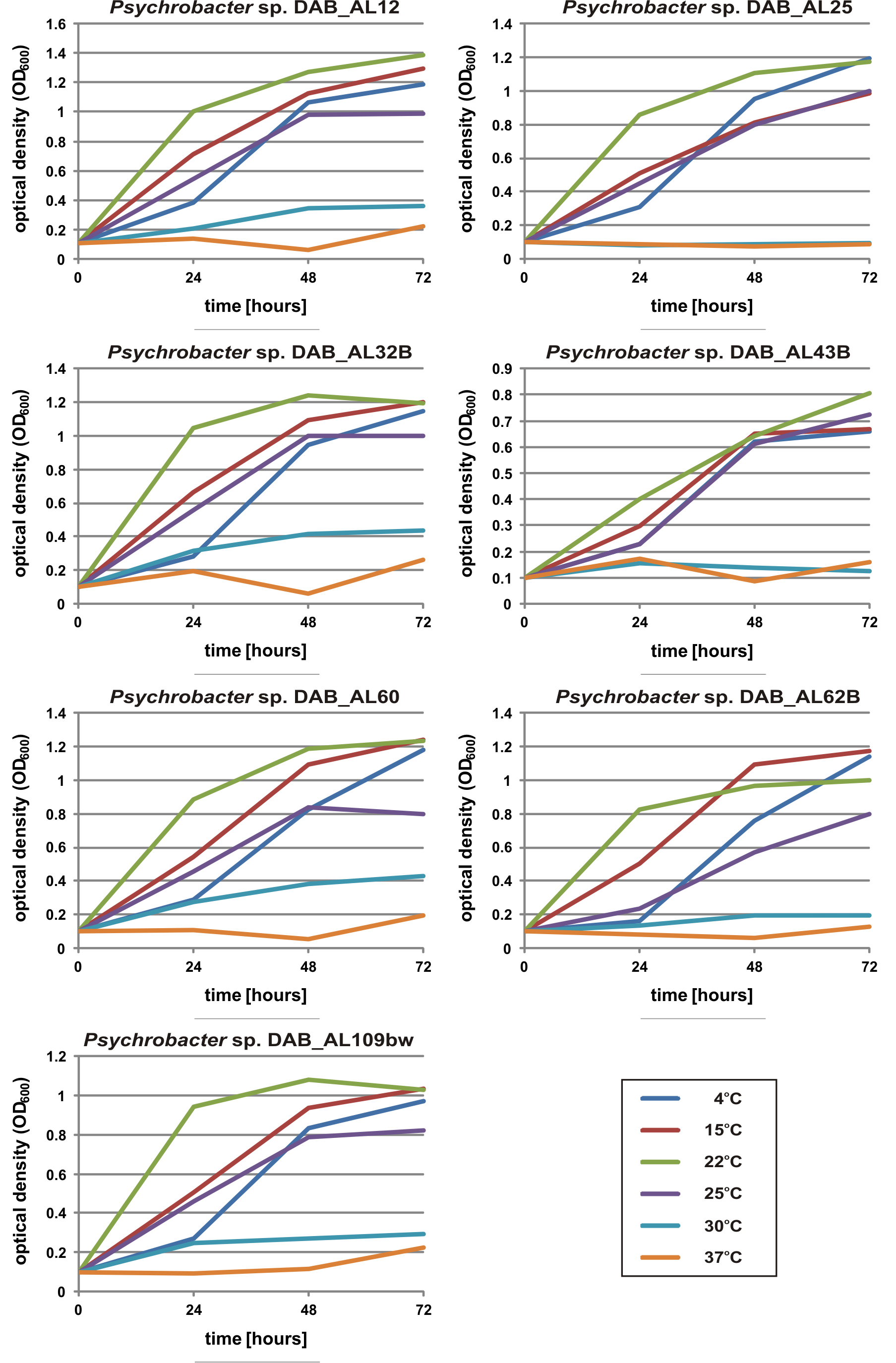

Supplement: Supplementary file 1 — Supplementary Figure S1 (TIFF 14570 kb) [file 792_2013_521_MOESM1_ESM.tif]

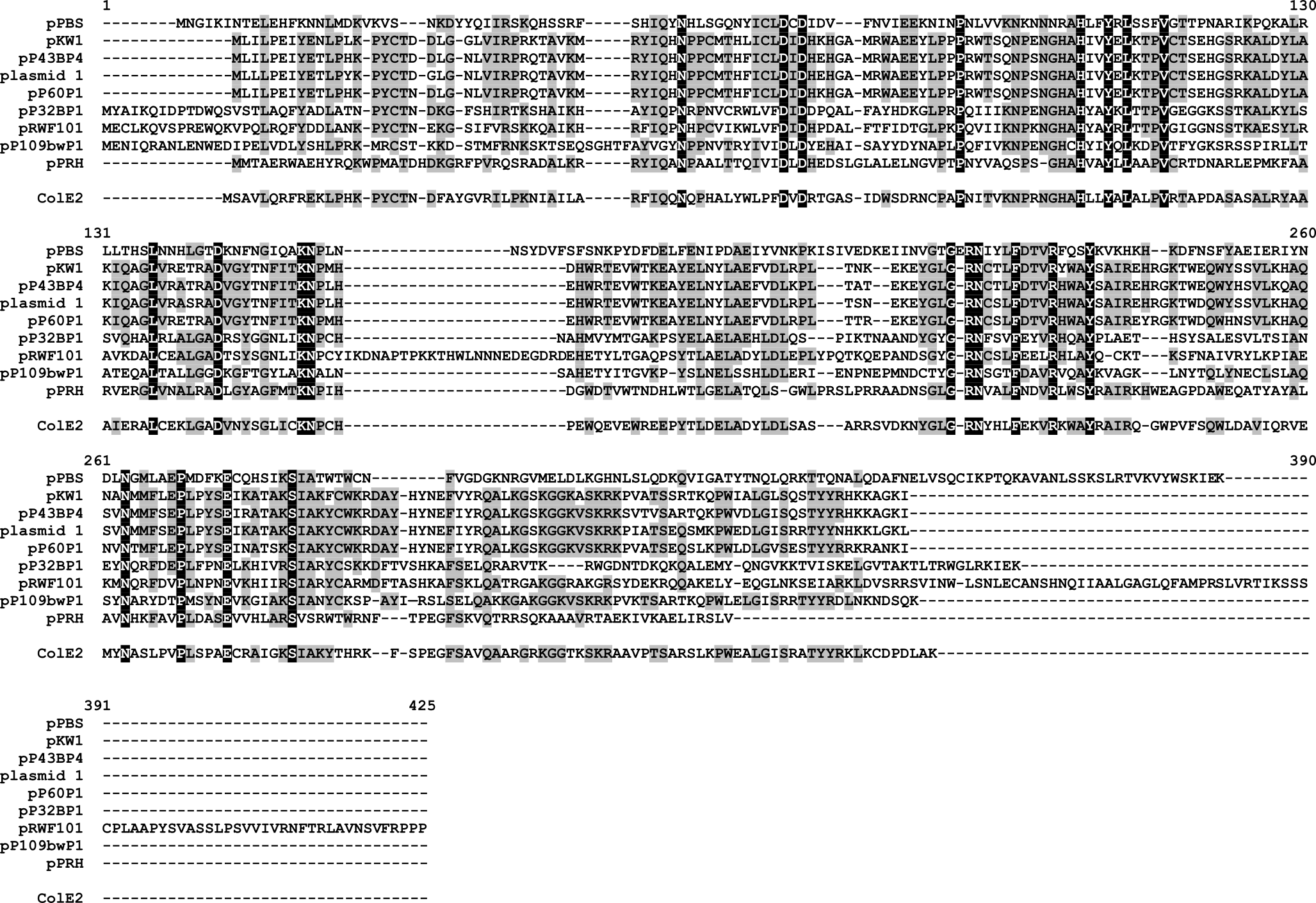

Supplement: Supplementary file 2 — Alignment of RepA-like replication proteins of the following plasmids: pPBS of Pseudoalteromonas sp. Bsi429; pKW1 of Pseudoalteromonas sp. 643A; pP43BP4 of Psychrobacter sp. DAB_AL43B; plasmid 1 of Psychrobacter cryohalolentis K5; pP60P1 of Psychrobacter sp. DAB_AL60; pP32BP2 of Psychrobacater sp. DAB_AL32B; pRWF101 of Psychrobacter sp. PRwf-1; pP109bwP1 of Psychrobacter sp. DAB_AL109bw; pPRH of Arthrobacter rhombi; with ColE2 of E. coli as a reference plasmid. For the accession numbers of particular plasmids, see Table S2 (Supplementary Materials). Identical (within all analyzed sequences) amino acids are shown against a black background and those common to at least 50% of the analyzed sequences have a gray background (TIFF 8501 kb) [file 792_2013_521_MOESM2_ESM.tif]

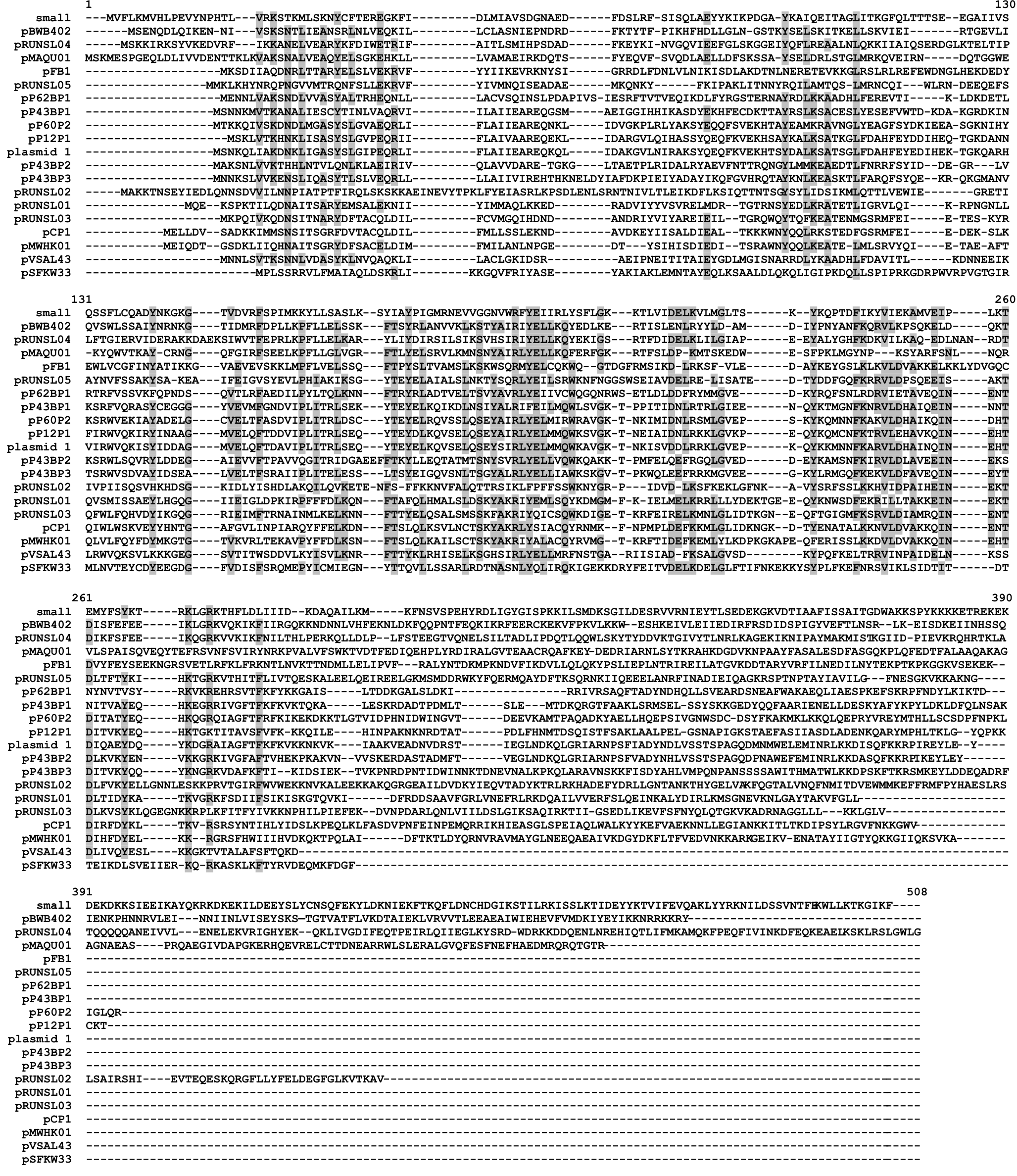

Supplement: Supplementary file 3 — Alignment of RepB-like replication proteins of the following plasmids: pBWB402 of Bacillus weihenstephanensis KBAB4; plasmid small of Desulfotalea psychrophila LSv54; pRUNSL01, pRUNSL02, pRUNSL03, pRUNSL04 and pRUNSL05 of Runella slithyformis DSM 19594; pMAQU01 of Marinobacter aquaeolei VT8; pFB1 of Flavobacterium branchiophilum FL-15; pP62BP1 of Psychrobacter sp. DAB_AL62B; pP43BP1, pP43BP2 and pP43BP3 of Psychrobacter sp. DAB_AL43B; pP60P2 of Psychrobacter sp. DAB_AL60; pP12P1 of Psychrobacter sp. DAB_AL12; plasmid 1 of Psychrobacter cryohalolentis K5; pCP1 of Flavobacterium psychrophilum D12; pMWHK01 of Pseudomonas sp. CG21; pVSAL43 of Allivibrio salmonicida LFI1238; and pSFKW33 of Shewanella sp. 33B. For the accession numbers of particular plasmids, see Table S2 (Supplementary Materials). Amino acids common to at least 50% of the analyzed sequences have a gray background (TIFF 14321 kb) [file 792_2013_521_MOESM3_ESM.tif]

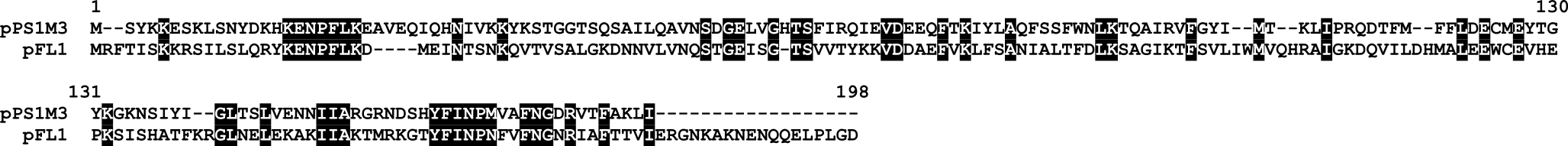

Supplement: Supplementary file 4 — Alignment of RepL-like replication proteins of the following plasmids: pPS1M3 of Pseudoalteromonas sp. PS1M3 and pFL1 of Flavobacterium sp. KP1. For accession numbers of particular plasmids, see Table S2 (Supplementary Materials). Identical (within both analyzed sequences) amino acids are shown against a black background (TIFF 1157 kb) [file 792_2013_521_MOESM4_ESM.tif]
