# Supplementary material for: Plasmid diversity in arctic strains of Psychrobacter spp
Source: Extremophiles. 2013 Mar 12;17(3):433–44. doi: 10.1007/s00792-013-0521-0 (PMC3632715; doi:10.1007/s00792-013-0521-0)
Supplement: Supplementary file 5 — Supplementary Table S1 (DOC 40 kb) [file 792_2013_521_MOESM5_ESM.doc]

**Table S1.** Cultivable bacteria isolated from the little auks guano.

| **Genera** | **Taxonomy** | **Strains** |
| --- | --- | --- |
| **gram-negative bacteria** | | |
| *Acinetobacter* | *Bacteria*; *Proteobacteria*; *Gammaproteobacteria*; *Pseudomonadales*; *Moraxellaceae* | DAB_ALA6 |
| *Chryseobacterium* | *Bacteria*; *Bacteroidetes*; *Flavobacteria*; *Flavobacteriales*; *Flavobacteriaceae* | DAB_AL8  DAB_AL45  DAB_AL113zr  DAB_ALA31  DAB_ALA45  DAB_ALK74 |
| *Flavobacterium* | *Bacteria*; *Bacteroidetes*; *Flavobacteria*; *Flavobacteriales*; *Flavobacteriaceae* | DAB_ALS1  DAB_AL27zr  DAB_AL29zr  DAB_AL30  DAB_AL32zr  DAB_AL42  DAB_AL44zr  DAB_AL50  DAB_AL71  DAB_AL114zr  DAB_ALA18  DAB_ALA19  DAB_ALA30  DAB_ALA32 |
| *Gelidibacter* | *Bacteria*; *Bacteroidetes*; *Flavobacteria*; *Flavobacteriales*; *Flavobacteriaceae* | DAB_AL58zr  DAB_AL59 |
| *Polaromonas* | *Bacteria*; *Proteobacteria*; *Betaproteobacteria*; *Burkholderiales*; *Comamonadaceae* | DAB_AL13Saa |
| *Pseudochrobactrum* | *Bacteria*; *Proteobacteria*; *Alphaproteobacteria*; *Rhizobiales*; *Brucellaceae* | DAB_ALK91 |
| *Pseudomonas* | *Bacteria*; *Proteobacteria*; *Gammaproteobacteria*; *Pseudomonadales*; *Pseudomonadaceae* | DAB_ALS2  DAB_ALA1  DAB_ALA4  DAB_ALA5  DAB_ALA21  DAB_ALA50  DAB_ALM1 |
| *Psychrobacter* | *Bacteria*; *Proteobacteria*; *Gammaproteobacteria*; *Pseudomonadales*; *Moraxellaceae* | DAB_AL4  DAB_AL6  DAB_AL12  DAB_AL18zr  DAB_AL25  DAB_AL32B  DAB_AL43B  DAB_AL49  DAB_AL60  DAB_AL62B  DAB_AL109bw  DAB_AL109bwb  DAB_AL109zr  DAB_ALK66  DAB_ALK87  DAB_ALK97  DAB_ALK99 |
| *Yersinia* | *Bacteria*; *Proteobacteria*; *Gammaproteobacteria*; *Enterobacteriales*; *Enterobacteriaceae* | DAB_ALA3  DAB_ALM52  DAB_ALM99 |
| **gram-positive bacteria** | | |
| *Arthrobacter* | *Bacteria*; *Actinobacteria*; *Actinobacteria*; *Actinobacteridae*; *Actinomycetales*; *Micrococcineae*; *Micrococcaceae* | DAB_AL9  DAB_AL55  DAB_AL28  DAB_AL115zr  DAB_ALK1  DAB_ALK1a  DAB_ALK1d  DAB_ALK4  DAB_ALK5  DAB_ALK10  DAB_ALK10a  DAB_ALK10c  DAB_ALK11  DAB_ALK15  DAB_ALK30  DAB_ALK34  DAB_ALK36  DAB_ALK38  DAB_ALK45  DAB_ALK47  DAB_ALK100  DAB_ALM15  DAB_ALM21  DAB_ALM26  DAB_ALM33  DAB_ALM36  DAB_ALM55  DAB_ALM62  DAB_ALM82  DAB_ALM94  DAB_ALM100  DAB_ALM101  DAB_ALM104  DAB_ALM106 |
| *Brevibacterium* | *Bacteria*; *Actinobacteria*; *Actinobacteria*; *Actinobacteridae*; *Actinomycetales*; *Micrococcineae*; *Brevibacteriaceae* | DAB_ALM2 |
| *Leifsonia* | *Bacteria*; *Actinobacteria*; *Actinobacteria*; *Actinobacteridae*; *Actinomycetales*; *Micrococcineae*; *Microbacteriaceae* | DAB_ALK95 |
