# Supplementary material for: Plasmid diversity in arctic strains of Psychrobacter spp
Source: Extremophiles. 2013 Mar 12;17(3):433–44. doi: 10.1007/s00792-013-0521-0 (PMC3632715; doi:10.1007/s00792-013-0521-0)
Supplement: Supplementary file 6 — Supplementary Table S2 (DOC 209 kb) [file 792_2013_521_MOESM6_ESM.doc]

Table S2. ORFs located within analyzed *Psychrobacter* plasmids.

| **ORFs located within plasmid pP12P1 of *Psychrobacter* sp. DAB_AL12** | | | | | | | |
| --- | --- | --- | --- | --- | --- | --- | --- |
| ORF no. | Coding region (bp) | Orientation | Protein size (aa) | Possible function | Best BLAST hits | | |
|  |  |  |  |  | Percentage identity (aa) | Organism | GenBank accession no. |
| 1 | 307-  1263 | ← | 318 | Initiator RepB protein | 74  (164/221) | *Psychrobacter cryohalolentis* K5  (plasmid 1) | YP_579255 |
| 2 | 2068-2607 | → | 179 | Hypothetical protein | 34  (48/141) | *Pantoea vagans* C9-1 | YP_003930687 |
| **ORFs located within plasmid pP32BP1 of *Psychrobacter* sp. DAB_AL32B** | | | | | | | |
| ORF no. | Coding region (bp) | Orientation | Protein size (aa) | Possible function | Best BLAST hits | | |
|  |  |  |  |  | Percentage identity (aa) | Organism | Coding region (bp) |
| 1 | 320-  1267 | → | 315 | Plasmid replicase  (RepA) | 53  (178/337) | *Psychrobacter sp.*  PRwf-1  (plasmid pRWF101) | YP_001274370 |
| 2 | 1697-1864 | → | 55 | YacA-like protein | 58  (30/52) | *Moraxella catarrhalis* RH4 | YP_003627349 |
| 3 | 1856-2125 | ← | 89 | Mobilization protein C (MobC) | 47  (35/74) | *Pseudomonas syringae* pv. maculicola ES4326 (plasmid pPMA4326C) | YP_025711 |
| 4 | 2324-3304 | → | 326 | Mobilization protein A (MobA) | 52  (135/262) | *Moraxella bovis*  ATCC 10900  (plasmid pMbo4.6) | YP_003289296 |
| 5 | 3301-3852 | → | 183 | Hypothetical protein | 31  (35/113) | *Acinetobacter lwoffii* SH145 | ZP_06071142 |
| 6 | 3925-4599 | → | 224 | Hypothetical protein, Sel1 domain protein | 51  (68/134) | *Moraxella catarrhalis* 12P80B1 | EGE16689 |
| **ORFs located within plasmid pP43BP1 of *Psychrobacter* sp. DAB_AL43B** | | | | | | | |
| ORF no. | Coding region (bp) | Orientation | Protein size (aa) | Possible function | Best BLAST hits | | |
|  |  |  |  |  | Percentage identity (aa) | Organism | Coding region (bp) |
| 1 | 286-  1233 | → | 315 | Initiator RepB protein | 45 (138/309) | *Acinetobacter* sp. SH024 | ZP_06693793 |
| 2 | 1281-1766 | → | 161 | Hypothetical protein | 84  (36/161) | *Psychrobacter arcticus* 273-4 | YP_264162 |
| 3 | 1849-2193 | ← | 114 | Hypothetical protein | - | No hits found | - |
| 4 | 2374-3423 | ← | 349 | Mobilization protein A (MobA) | 46  (175/379) | *Psychrobacter sp.* 1501(2011) | ZP_08462213 |
| 5 | 3420-3740 | ← | 106 | Mobilization protein C (MobC) | 59  (57/97) | *Psychrobacter sp.*  PRwf-1  (plasmid pRWF101) | YP_001274382 |
| 6 | 3986-4390 | → | 134 | Hypothetical protein | 36  (40/111) | *Psychrobacter sp.*  PRwf-1  (plasmid pRWF101) | YP_001274369 |
| **ORFs located within plasmid pP43BP2 of *Psychrobacter* sp. DAB_AL43B** | | | | | | | |
| ORF no. | Coding region (bp) | Orientation | Protein size (aa) | Possible function | Best BLAST hits | | |
|  |  |  |  |  | Percentage identity (aa) | Organism | Coding region (bp) |
| 1 | 498-  1445 | → | 315 | Initiator RepB protein | 61 (150/245) | *Acinetobacter johnsonii* SH046 | ZP_06064826 |
| 2 | 1612-2607 | → | 331 | Hypothetical protein | 35  (105/301) | *Nitrosomonas sp.* AL212 | YP_004294730 |
| 3 | 3169-3450 | → | 93 | Hypothetical protein | - | No hits found | - |
| 4 | 3461-3664 | ← | 67 | RelB-like antitoxin of toxin-antitoxin module | 96  (51/53) | *Psychrobacter cryohalolentis* K5  (plasmid 1) | YP_579251 |
| 5 | 3729-4046 | ← | 109 | Hypothetical protein | 38  (38/100) | *Psychrobacter cryohalolentis* K5  (plasmid 1) | YP_579252 |
| 6 | 4504-5445 | → | 313 | Mobilization protein A (MobA) | 48  (135/279) | *Acinetobacter baumannii*  135040 | ACX70401 |
| **ORFs located within plasmid pP43BP3 of *Psychrobacter* sp. DAB_AL43B** | | | | | | | |
| ORF no. | Coding region (bp) | Orientation | Protein size (aa) | Possible function | Best BLAST hits | | |
|  |  |  |  |  | Percentage identity (aa) | Organism | Coding region (bp) |
| 1 | 446-  1438 | → | 330 | Initiator RepB protein | 57 (147/257) | *Acinetobacter sp.* SH024 | ZP_06693793 |
| 2 | 1596-2144 | ← | 182 | Hypothetical protein, YebN family protein | 77 (141/182) | *Psychrobacter cryohalolentis* K5 | YP_580604 |
| 3 | 2231-2563 | → | 110 | Hypothetical protein | 100 (110/110) | *Psychrobacter cryohalolentis* K5 | YP_580606 |
| 4 | 2656-2922 | ← | 88 | RelE-like antitoxin of toxin-antitoxin module | 67  (58/86) | *Cardiobacterium valvarum* F0432 | ZP_09445945 |
| 5 | 2909-3190 | ← | 93 | RelB-like antitoxin of toxin-antitoxin module | 67  (41/61) | *Psychrobacter cryohalolentis* K5  (plasmid 1) | YP_579251 |
| 6 | 3281-3556 | ← | 91 | Hypothetical protein | 42  (36/86) | *Psychrobacter cryohalolentis* K5  (plasmid 1) | YP_579252 |
| 7 | 4014-4955 | → | 313 | Mobilization protein A (MobA) | 47  (132/279) | *Acinetobacter baumannii*  135040 | ACX70401 |
| **ORFs located within plasmid pP43BP4 of *Psychrobacter* sp. DAB_AL43B** | | | | | | | |
| ORF no. | Coding region (bp) | Orientation | Protein size (aa) | Possible function | Best BLAST hits | | |
|  |  |  |  |  | Percentage identity (aa) | Organism | Coding region (bp) |
| 1 | 454-  1335 | → | 293 | Plasmid replicase  (RepA) | 91  (267/293) | *Psychrobacter cryohalolentis* K5  (plasmid 1) | YP_579243 |
| 2 | 1520-1831 | ← | 103 | Hypothetical protein | 36  (27/74) | *Paenibacillus lactis* 154 | ZP_09002102 |
| 3 | 1853-2311 | ← | 152 | Hypothetical protein | 55  (56/102) | *Psychrobacter cryohalolentis* K5 | YP_581222 |
| 4 | 2425-2892 | ← | 155 | Hypothetical protein | 75  (117/157) | *Psychrobacter arcticus* 273-4 | YP_263495 |
| 5 | 3187-3519 | → | 110 | Mobilization protein C (MobC) | 47  (51/108) | *Pseudoalteromonas sp. 643A*  (plasmid pKW1) | YP_001887741 |
| 6 | 3509-5422 | → | 637 | Mobilization protein A (MobA) | 64  (144/224) | *Pseudoalteromonas sp. 643A*  (plasmid pKW1) | YP_001887742 |
| 7 | 4221-4772 | → | 183 | Hypothetical protein | 35  (54/153) | *Pseudoalteromonas sp. 643A*  (plasmid pKW1) | YP_001887743 |
| 8 | 4776-5348 | → | 190 | Hypothetical protein | 36  (68/187) | *Pseudoalteromonas sp. 643A*  (plasmid pKW1) | YP_001887745 |
| 9 | 5596-6450 | → | 284 | Hypothetical protein | 39 (64/163) | Gramella forsetii KT0803 | YP_862674 |
| **ORFs located within plasmid pP60P1 of *Psychrobacter* sp. DAB_AL60** | | | | | | | |
| ORF no. | Coding region (bp) | Orientation | Protein size (aa) | Possible function | Best BLAST hits | | |
|  |  |  |  |  | Percentage identity (aa) | Organism | Coding region (bp) |
| 1 | 283-  1164 | → | 293 | Plasmid replicase  (RepA) | 90 (263/293) | *Psychrobacter cryohalolentis* K5  (plasmid 1) | YP_579243 |
| 2 | 1240-1785 | ← | 181 | Hypothetical protein | - | No hits found | - |
| 3 | 1836-2234 | ← | 132 | Hypothetical protein | - | No hits found | - |
| 4 | 2536-2868 | → | 110 | Mobilization protein C (MobC) | 46  (50/108) | *Pseudoalteromonas sp. 643A*  (plasmid pKW1) | YP_001887741 |
| 5 | 2858-4771 | → | 637 | Mobilization protein A (MobA) | 66  (138/209) | *Pseudoalteromonas sp. 643A*  (plasmid pKW1) | YP_001887742 |
| 6 | 3570-4121 | → | 183 | Hypothetical protein | 32  (49/153) | *Pseudoalteromonas sp. 643A*  (plasmid pKW1) | YP_001887743 |
| 7 | 4125-4700 | → | 191 | Hypothetical protein | 36  (68/187) | *Pseudoalteromonas sp. 643A*  (plasmid pKW1) | YP_001887745 |
| 8 | 4959-5435 | → | 158 | Hypothetical protein | 45  (72/160) | uncultured bacterium clone BF11_C10 | ACN22658 |
| **ORFs located within plasmid pP60P2 of *Psychrobacter* sp. DAB_AL60** | | | | | | | |
| ORF no. | Coding region (bp) | Orientation | Protein size (aa) | Possible function | Best BLAST hits | | |
|  |  |  |  |  | Percentage identity (aa) | Organism | Coding region (bp) |
| 1 | 344-  1333 | → | 329 | Initiator RepB protein | 56 (161/290) | *Psychrobacter cryohalolentis* K5  (plasmid 1) | YP_579255 |
| 2 | 1801-1992 | ← | 63 | Transposase | 72  (39/54) | *Psychrobacter cryohalolentis* K5 | YP_580194 |
| 3 | 2109-2603 | ← | 164 | Hypothetical protein | 71  (119/168) | *Psychrobacter sp.* J466 | ACY02902 |
| 4 | 2937-3734 | → | 265 | Hypothetical protein, Sel1 domain protein | 46  (102/221) | *Candidatus* Amoebophilus asiaticus  5a2 | YP_001957952 |
| 5 | 4271-5683 | → | 470 | DNA (cytosine-5-)-methyltransferase | 60  (255/424) | *Marivirga tractuosa* DSM 4126 | YP_004054233 |
| 6 | 5727-6626 | ← | 299 | Hypothetical protein | 47  (135/290) | *Moraxella catarrhalis* BC1 | EGE16233 |
| 7 | 6883-7449 | → | 188 | Alkyl hydroperoxide reductase, subunit C | 99 (187/188) | *Psychrobacter arcticus* 273-4 | YP_265079 |
| 8 | 8258-9298 | → | 346 | Hypothetical protein, Sel1 domain protein | 48 (158/331) | *Neisseria sp.* GT4A_CT1 | ZP_08890114 |
| 9 | 9413-9865 | → | 150 | Hypothetical protein | 32  (33/103) | Enhydrobacter aerosaccus SK60 | ZP_05621186 |
| 10 | 9909-10529 | ← | 206 | Resolvase | 83 (169/204) | *Acinetobacter baumannii* 6013150 | ZP_08433422 |
| 11 | 10816-11850 | → | 344 | ABC transporter ATP-binding protein | 31  (34/108) | *Lactobacillus plantarum* JDM1 | YP_003064009 |
| 12 | 11989-12504 | ← | 171 | Mobilization protein C (MobC) | 36  (42/116) | *Psychrobacter* sp. 1501(2011) | ZP_08462219 |
| 13 | 12792-14924 | → | 710 | Mobilization protein A (MobA) | 77  (550/712) | *Psychrobacter sp.* J466 | ACY02904 |
| **ORFs located within plasmid pP109bwP1 of *Psychrobacter* sp. DAB_AL109bw** | | | | | | | |
| ORF no. | Coding region (bp) | Orientation | Protein size (aa) | Possible function | Best BLAST hits | | |
|  |  |  |  |  | Percentage identity (aa) | Organism | Coding region (bp) |
| 1 | 218-1156 | → | 312 | Plasmid replicase  (RepA) | 39 (113/293) | *Moraxella catarrhalis*  (plasmid pEMCJH03) | NP_957539 |
| 2 | 1414-1578 | ← | 54 | RelB-like antitoxin of addiction module | 45  (18/40) | *Desulfitobacterium hafniense* DCB-2 | YP_002460026 |
| 3 | 2239-2571 | → | 110 | Mobilization protein C (MobC) | 58  (63/108) | *Pseudoalteromonas sp. 643A*  (plasmid pKW1) | YP_001887741 |
| 4 | 2561-4402 | → | 613 | Mobilization protein A (MobA) | 61  (137/223) | *Pseudoalteromonas sp. 643A*  (plasmid pKW1) | YP_001887742 |
| 5 | 3273-3824 | → | 183 | Hypothetical protein | 35  (53/153) | *Pseudoalteromonas sp. 643A*  (plasmid pKW1) | YP_001887743 |
| 6 | 3828-4346 | → | 172 | Hypothetical protein | 35  (60/173) | *Pseudoalteromonas sp. 643A*  (plasmid pKW1) | YP_001887745 |
