# Supplementary material for: Plasmid diversity in arctic strains of Psychrobacter spp
Source: Extremophiles. 2013 Mar 12;17(3):433–44. doi: 10.1007/s00792-013-0521-0 (PMC3632715; doi:10.1007/s00792-013-0521-0)
Supplement: Supplementary file 7 — Supplementary Table S3 (DOC 50 kb) [file 792_2013_521_MOESM7_ESM.doc]

Table S3. Plasmids of psychrophilic bacteria.

| **Organism** | **Plasmid** | **Plasmid acc. no.** |
| --- | --- | --- |
| *Allivibrio salmonicida* LFI1238 | pVSAL320  pVSAL43  pVSAL54  pVSAL840 | FM178382  FM178384  FM178383  FM178381 |
| *Arthrobacter rhombi* | pPRH | NC_016615 |
| *Bacillus weihenstephanensis* KBAB4 | pBWB401  pBWB402  pBWB403  pBWB404 | NC_010180  NC_010181  NC_010182  NC_010183 |
| *Desulfotalea psychrophila* LSv54 | plasmid small  plasmid large | NC_006140  NC_006139 |
| *Exiguobacterium sibiricum* 255-15 | pEXIG01  pEXIG02 | CP001023  CP001024 |
| *Flavobacterium branchiophilum* FL-15 | pFB1 | FQ859182 |
| *Flavobacterium psychrophilum* D12 | pCP1 | AY277637 |
| *Flavobacterium* sp. KP1 | pFL1 | NC_002132 |
| *Glaciecola* sp. 4H-3-7+YE-5 | pGLAAG01 | NC_015498 |
| *Marinobacter aquaeolei* VT8 | pMAQU01  pMAQU02 | NC_008738  NC_008739 |
| *Photobacterium profundum* SS9 | plasmid 1 | CR377818 |
| *Pseudoalteromonas* sp. 643A | pKW1 | NC_010675 |
| *Pseudoalteromonas* sp. BSi20327 | pSM327 | GU198194 |
| *Pseudoalteromonas sp.* Bsi429 | pPBS | EU627679 |
| *Pseudoalteromonas* sp. PS1M3 | pPS1M3 | AB022096 |
| *Pseudomonas* sp. CG21 | pMWHK01 | FJ613505 |
| *Pseudomonas* sp. MC1 | plasmid KOPRI126573 | NC_016644 |
| *Psychrobacter cryohalolentis* K5 | plasmid 1 | NC_007968 |
| *Psychrobacter* sp. DAB_AL109bw | pP109bwP1 | JQ245702 |
| *Psychrobacter* sp. DAB_AL12 | pP12P1 | JQ231228 |
| *Psychrobacter* sp. DAB_AL32B | pP32BP1 | JQ245699 |
| *Psychrobacter* sp. DAB_AL43B | pP43BP1  pP43BP2  pP43BP3  pP43BP4 | JQ245700  JQ245701  JQ348845  JQ348844 |
| *Psychrobacter* sp. DAB_AL60 | pP60P1  pP60P2 | JQ245703  JQ245704 |
| *Psychrobacter* sp. DAB_AL62B | pP62BP1 | JQ065022 |
| *Psychrobacter* sp. PRwf-1 | pRWF101  pRWF102 | NC_009516  NC_009517 |
| *Runella slithyformis* DSM 19594 | pRUNSL01  pRUNSL02  pRUNSL03  pRUNSL04  pRUNSL05 | CP002860  CP002861  CP002862  CP002863  CP002864 |
| *Shewanella* sp. 33B | pSFKW33 | FJ626843 |
